# Synuclein Regulates Synaptic Vesicle Clustering and Docking at a Vertebrate Synapse

**DOI:** 10.3389/fcell.2021.774650

**Published:** 2021-11-26

**Authors:** Kaitlyn E. Fouke, M. Elizabeth Wegman, Sarah A. Weber, Emily B. Brady, Cristina Román-Vendrell, Jennifer R. Morgan

**Affiliations:** ^1^ The Eugene Bell Center for Regenerative Biology and Tissue Engineering, Marine Biological Laboratory, Woods Hole, MA, United States; ^2^ Department of Neurobiology, Duke University, Durham, NC, United States; ^3^ Biological Sciences Collegiate Division, The University of Chicago, Chicago, IL, United States

**Keywords:** exocytosis, endocytosis, synapsin, lamprey, liquid phase separation, VAMP2

## Abstract

Neurotransmission relies critically on the exocytotic release of neurotransmitters from small synaptic vesicles (SVs) at the active zone. Therefore, it is essential for neurons to maintain an adequate pool of SVs clustered at synapses in order to sustain efficient neurotransmission. It is well established that the phosphoprotein synapsin 1 regulates SV clustering at synapses. Here, we demonstrate that synuclein, another SV-associated protein and synapsin binding partner, also modulates SV clustering at a vertebrate synapse. When acutely introduced to unstimulated lamprey reticulospinal synapses, a pan-synuclein antibody raised against the N-terminal domain of α-synuclein induced a significant loss of SVs at the synapse. Both docked SVs and the distal reserve pool of SVs were depleted, resulting in a loss of total membrane at synapses. In contrast, antibodies against two other abundant SV-associated proteins, synaptic vesicle glycoprotein 2 (SV2) and vesicle-associated membrane protein (VAMP/synaptobrevin), had no effect on the size or distribution of SV clusters. Synuclein perturbation caused a dose-dependent reduction in the number of SVs at synapses. Interestingly, the large SV clusters appeared to disperse into smaller SV clusters, as well as individual SVs. Thus, synuclein regulates clustering of SVs at resting synapses, as well as docking of SVs at the active zone. These findings reveal new roles for synuclein at the synapse and provide critical insights into diseases associated with α-synuclein dysfunction, such as Parkinson’s disease.

## Introduction

Neurotransmission depends on the rapid, efficient release of neurotransmitters from small synaptic vesicles (SVs), which are maintained in tight clusters at the presynaptic active zone (Pang and Sudhof, 2010). Functionally, the SV cluster is organized into two pools: the readily releasable pool (RRP) of SVs docked at the active zone membrane, and the reserve pool of distal SVs that comprise the majority of the vesicle cluster (Rizzoli and Betz, 2004, 2005; Denker and Rizzoli, 2010; Chanaday et al., 2019). Upon synaptic stimulation, the RRP is the first to undergo exocytosis and neurotransmitter release, whereas the reserve pool is mobilized during sustained levels of synaptic activity only after the RRP is depleted (Pieribone et al., 1995; Rosenmund and Stevens, 1996). Subsequently, the SVs are locally recycled via endocytosis, refilled with neurotransmitters, and re-clustered for use in subsequent bouts of neurotransmitter release (Sudhof, 2004; Saheki and De Camilli, 2012; Chanaday et al., 2019). Maintaining SV clusters is therefore essential for neurotransmission and neural function. Indeed, many neurodegenerative diseases are associated with dysfunctional synapses, including a loss of SVs and neurotransmission deficits.

SV clustering is regulated by the synaptic vesicle-associated phosphoprotein, synapsin (De Camilli et al., 1983a; De Camilli et al., 1983b; Cesca et al., 2010; Longhena et al., 2021; Zhang and Augustine, 2021). A pioneering study at the lamprey reticulospinal (RS) synapse demonstrated that acute disruption of synapsin I with inhibitory antibodies caused a complete loss of the distal pool of SVs, leaving only docked SVs intact (Pieribone et al., 1995). As a consequence, synapsin inhibition caused a rapid run-down of synaptic transmission during high frequency stimulation (Pieribone et al., 1995). These findings have since been corroborated in synapse models ranging from the invertebrate squid giant synapse to mammalian hippocampal synapses (Hilfiker et al., 1999; Gitler et al., 2008; Pechstein et al., 2020). According to the classical view of SV clustering, synapsin cross-links SVs together in a “scaffold” of protein-protein interactions between synapsin and its binding partners, such as actin (Cesca et al., 2010; Zhang and Augustine, 2021). Recent *in vitro* studies, however, have instead proposed that synapsin clusters SVs via liquid-liquid phase separation (LLPS) through synapsin’s intrinsically-disordered regions, by forming synapsin-SV condensates that are separated from the surrounding buffer (Milovanovic and De Camilli, 2017; Hoffmann et al., 2021; Park et al., 2021). A recent *in vivo* study at lamprey synapses supports this new model (Pechstein et al., 2020). That synapsin may cluster SVs via an LLPS mechanism has transformed our understanding of SV clustering. However, the scaffolding versus LLPS models for SV clustering are not necessarily mutually-exclusive and are still under debate, as are the molecular mechanisms (Zhang and Augustine, 2021).

Here, we examined whether α-synuclein also plays a role in SV clustering since this protein appears to cooperate functionally with synapsin at synapses (Atias et al., 2019). α-Synuclein is another SV-associated presynaptic protein that regulates SV trafficking (Maroteaux and Scheller, 1991; George, 2002; Sulzer and Edwards, 2019). Although its normal, physiological functions are still under investigation, current data support roles for α-synuclein in several stages of exocytosis and endocytosis, including: SNARE complex formation, fusion pore dilation, and early stages of clathrin-mediated vesicle endocytosis (Burre et al., 2010; Greten-Harrison et al., 2010; Vargas et al., 2014; Logan et al., 2017; Sulzer and Edwards, 2019). α-Synuclein may therefore be a multi-functional regulator of SV trafficking at synapses (Bendor et al., 2013; Sulzer and Edwards, 2019). A recent study reported that α-synuclein co-condensated along with synapsin 1 *in vitro* and in cells upon ectopic expression, raising the interesting possibility that α-synuclein regulates SV clustering either alone or in coordination with synapsin (Hoffmann et al., 2021). In addition, direct and genetic interactions between α-synuclein and synapsin have been reported (Nemani et al., 2010; Scott et al., 2010; Atias et al., 2019), and α-synuclein regulates SV pool dynamics (Scott and Roy, 2012). However, while standard genetic manipulations of α−synuclein (i.e. chronic overexpression or knockout) have revealed many key features of α-synuclein function, these manipulations are also known to alter expression levels and phosphorylation status of synapsin and several other major presynaptic proteins (Nemani et al., 2010; Scott et al., 2010; Vargas et al., 2014; Vargas et al., 2017). Thus, with chronic manipulations of α-synuclein, it can be difficult to assess which aspects of the synaptic phenotypes are directly mediated by α-synuclein or via compensatory molecular changes in other presynaptic proteins such as synapsin.

We therefore utilized an acute perturbation strategy to disrupt synuclein function at lamprey RS synapses. Microinjection of lamprey axons with an anti-pan-synuclein antibody induced a dose-dependent loss of SVs within the distal SV clusters representative of the reserve pool. Docked SVs were also depleted. The SVs appeared to de-cluster in a piecewise fashion into smaller SV clusters, consistent with an LLPS mechanism, as well as individual SVs. These results demonstrate that synuclein is a modulator of SV clustering and docking at resting synapses, thereby revealing novel functions of synucleins at living synapses.

## Methods

### Synuclein Structure Analysis

A multiple sequence alignment of human α-, β-, γ- and lamprey γ-synucleins was created using the msa package in R software. The GenBank accession numbers for the sequences used in the alignment were: human α-synuclein (NM_000345.4); human β-synuclein (NM_001001502.3); human γ-synuclein (NM_003087.3); and lamprey γ-synuclein (JN544525.1). Disorder probability and charge distribution analyses were performed using the PrDOS bioinformatics tool and EMBOSS package, respectively.

### Western Blotting

Standard Western blotting procedures were performed as previously described (Busch et al., 2014). For all experiments, 10 μg of rat brain lysates and 20 μg of lamprey CNS (brain and spinal cord) lysates were separated on 10% or 12% SDS-PAGE gels. Primary antibodies were as follows: anti-pan synuclein rabbit polyclonal antibody raised against amino acids 11-26 within the N-terminal domain of human α-synuclein (1:1000; ab6176; Abcam, Cambridge, MA); an anti-synaptobrevin (VAMP) mouse monoclonal antibody (1:1000; 1933-SYB, clone SP10, PhosphoSolutions, Aurora, CO); and an anti-SV2 mouse monoclonal antibody (1:1000; Developmental Studies Hybridoma Bank; University of Iowa, Iowa City, IA, United States). The SV2 antibody was deposited to the DSHB by Buckley, K.M., and it labels SV clusters in all vertebrates tested, including lampreys (DSHB Hybridoma Product SV2) (Buckley and Kelly, 1985; Lau et al., 2011; Busch and Morgan, 2012; Busch et al., 2014). Secondary antibodies were goat anti-rabbit or goat anti-mouse HRP conjugated IgG (H + L), as appropriate (Thermo Scientific, Waltham, MA, United States). Protein bands were detected using Pierce™ ECL Western blotting substrate (Thermo Scientific, Waltham, MA, United States) and imaged using an Azure Imaging System 300 (Azure Biosystems; Dublin, CA, United States).

### Microinjections and Electron Microscopy

Animal procedures were approved by the Institutional Animal Care and Use Committee at the MBL in accordance with standards set by the National Institutes of Health. Late larval lampreys (*Petromyzon marinus*; 11–13 cm; 5–7 years old; M/F) were anesthetized in 0.1–0.2 g/ L Tricaine-S (Syndel; Ferndale, WA, United States). Segments of spinal cord (2–3 cm) were dissected, pinned ventral side up in a Sylgard petri dish, and stripped of meninx. Axonal microinjections were performed as previously described (Busch et al., 2014; Walsh et al., 2018; Banks et al., 2020; Soll et al., 2020; Román-Vendrell et al., 2021). The antibodies injected included an anti-pan-synuclein antibody (ab6176; Abcam), anti-SV2 antibody (DSHB); and anti-synaptobrevin (VAMP) antibody (1933-SYB/SP10, PhosphoSolutions). Rabbit polyclonal IgGs (ab37415; Abcam) were also injected in order to provide a negative, isotype control for the synuclein antibody. Prior to injection, all antibodies were diluted in lamprey internal solution (180 mM KCl and 10 mM HEPES K^+^; pH 7.4) to a final pipet concentration of 0.5 mg/ml and mixed with fluorescein dextran (0.1 mM; 70 kDa; Thermo Fisher) in order to monitor the injections in real time. Antibodies were then loaded into glass microelectrodes and microinjected into giant RS axons using small, repeated pulses of N_2_ (5–30 ms, 40 psi, 0.2–0.3 Hz) delivered through a Toohey spritzer. Injections lasted 15–20 min and typically resulted in a 20-100x dilution of the antibody within the axon, based on the fluorescence of the co-injected dye. After injection, the spinal cords were immediately fixed in 3% glutaraldehyde, 2% paraformaldehyde in 0.1 M Na cacodylate, pH 7.4 overnight.

After fixation, spinal cords were processed for electron microscopy, thin sectioned at ∼70 nm, and counterstained with 2% uranyl acetate and 0.4% lead citrate, as detailed previously (Busch et al., 2014; Walsh et al., 2018; Banks et al., 2020; Soll et al., 2020; Román-Vendrell et al., 2021). Ultrastructural images were obtained at ×37,000 magnification using a JEOL JEM 200CX electron microscope. Serial images of giant reticulospinal synapses were acquired from at least *n* = 20 synapses, from 2-3 axons/lampreys for each experimental condition. Synapses were analyzed from three different regions of the injected axon (representing different concentration ranges), based on axonal diffusion patterns of the fluorescein dextran: >400 μm from the injection site, beyond where the injected protein had diffused (Untreated Control); 150–390 μm from the injection site (Low concentration); and 30–140 μm from the injection site (High concentration). Each experiment was therefore internally controlled, reducing variance due to natural differences in SV clusters between axons and animals.

Morphometric analysis was performed on a single image per synapse, taken at or near the center of the active zone, as previously described (Busch et al., 2014; Walsh et al., 2018; Banks et al., 2020; Soll et al., 2020; Román-Vendrell et al., 2021). These included synaptic vesicles (SVs), plasma membrane, cisternae (putative endosomes), as well as clathrin-coated pits (CCPs) and clathrin-coated vesicles (CCVs). SVs were defined as small, clear round vesicles <100 nm in diameter, while “cisternae” were defined as larger vesicles that were >100 nm in diameter. Plasma membrane evaginations were determined by drawing a straight 1 μm line from the edge of the active zone to the nearest position on the axolemma on both sides of the synapse, then measuring the curved distance between these points, and averaged. CCPs and CCVs were staged as detailed previously (Morgan et al., 2004). After obtaining measurements for each organelle, a total membrane analysis was performed on each synapse to determine if and how synaptic membranes were redistributed under each experimental condition. SV and CCP/V membrane areas were calculated by multiplying the surface area of a sphere (4πr^2^) by the number of each type of vesicle at each synapse. Membrane areas associated with plasma membrane and cisternae were obtained by multiplying the length of plasma membrane evaginations and summed cisternae perimeters, respectively, by the section thickness (70 nm). In addition, the SV distribution and nearest neighbor analysis were determined using a Python script (based on script in Banks et al., 2020; https://github.com/kfouke/Morgan-Lab), which measured the distance from the center of each SV to the nearest point on the active zone and to the center of the nearest SV. Graphing and statistical analyses, including ANOVA and linear regressions, were performed in GraphPad Prism 9.0.

## Results

### Lamprey Synuclein is Highly Conserved and Shares Similar Structural Features with Human α-Synuclein

**FIGURE 1:**
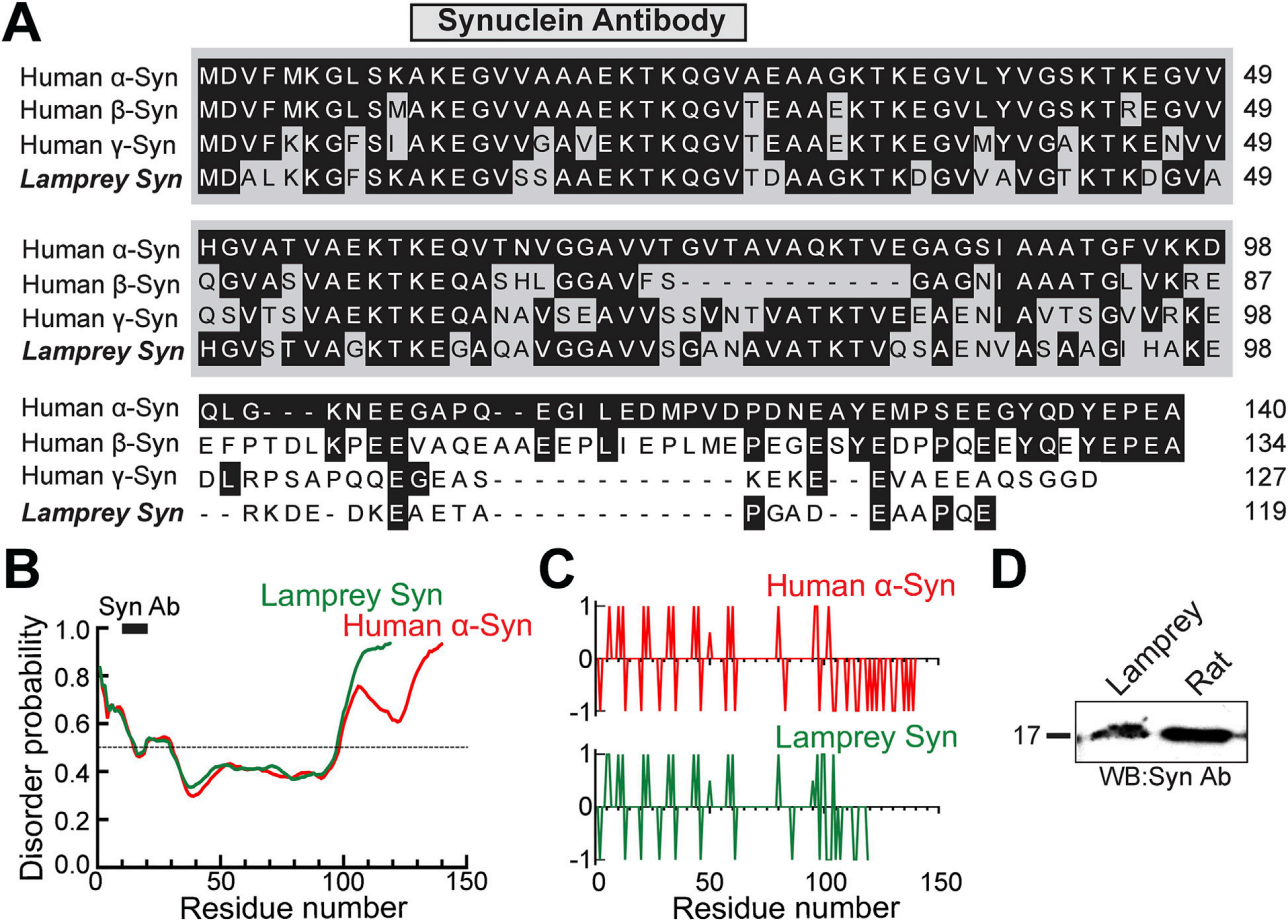
Lamprey and human synucleins are highly conserved. **(A)** Multiple sequence alignment of human synuclein (α, β, γ) and lamprey γ-synuclein (GenBank (JN544525.1). Black boxes indicate amino acid residues that are identical to human α-synuclein. The N-terminal domains (gray) are the most conserved sequences amongst synucleins. The epitope of the synuclein antibody used in this study is indicated (anti-pan-synuclein, ab6176 Abcam). **(B)** Disorder probability of human α-synuclein and lamprey γ-synuclein are nearly identical, even though the lamprey sequence is shorter. Sequences above the 0.5 probability threshold are predicted to be disordered, while those below are structured. Graphs were generated using PrDOS. Black bar indicates the Synuclein Ab epitope. **(C)** The charge distributions of human α-synuclein and lamprey γ-synuclein proteins are also nearly identical. Positively- and negatively-charged residues are indicated by + 1 and−1, respectively. Plots were generated using EMBOSS. **(D)** The pan-synuclein antibody, which was raised against the N-terminal domain of human α-synuclein, recognizes monomeric synuclein in both rat brain and lamprey CNS lysates.

The goal of this study was to determine whether synuclein modulates SV clustering at resting synapses, given its known interactions with synapsin. Mammals, including humans, express three isoforms of synuclein: α-synuclein, β-synuclein, and γ-synuclein. As in mammals, lampreys, which are jawless vertebrates, also express three isoforms of synuclein: two γ-synucleins (as observed in other fishes) and a third synuclein isoform that remains unassigned because it did not reliably group in a phylogenetic analysis with other α-, β-, or γ-synuclein orthologs (Busch and Morgan, 2012; Smith et al., 2013). Our prior study demonstrated that the most abundant isoform expressed within lamprey giant RS neurons is a γ-synuclein (GenBank: JN544525), whereas the other two synuclein isoforms were expressed at low or undetectable levels (Busch and Morgan, 2012). We therefore focus our analysis on this abundant γ-synuclein isoform and refer the reader to our prior study for details on the other lamprey synuclein isoforms (Busch and Morgan, 2012). The primary amino acid sequence of lamprey γ-synuclein is 56, 54, and 55% identical to full length human α-, β-, and γ-synuclein, respectively (Figure 1A). The highly conserved N-terminal domain of synuclein (a.a. 1-95), which folds into an amphipathic α-helix and contains the non-amyloid component (NAC; a. a. 60-95), is 66% identical and 78% similar between human α-synuclein and lamprey γ-synuclein orthologs (Figure 1A). In contrast, the C-termini are more variable between all synuclein orthologs. We previously reported the predicted structure of lamprey γ-synuclein, which is an N-terminal α-helix followed by a less structured random coil at the C-terminus, similar to the structure of human α-synuclein bound to lipid micelles (Ulmer et al., 2005; Busch and Morgan, 2012).

To further compare the structures of human α-synuclein and lamprey γ-synuclein, we ran disordered region and charge distribution predictions on their sequences using PrDOS (https://prdos.hgc.jp/cgi-bin/top.cgi) and EMBOSS (https://www.bioinformatics.nl/cgi-bin/emboss/charge), respectively. Although the lamprey synuclein protein is shorter in length, the protein disorder prediction analysis revealed nearly identical disorder probability between lamprey γ-synuclein and human α-synuclein (Figure 1B). Both orthologs possess intrinsically-disordered regions (IDRs) at their C-termini (Figure 1B). Similarly, the charge distributions across human α-synuclein and lamprey γ-synuclein proteins are nearly identical (Figure 1C). As another indicator of conservation, a pan-synuclein antibody (ab6176; Abcam) raised against a peptide in the N-terminal domain of human α-synuclein (a.a. 11-26) recognized monomeric synuclein in both lamprey CNS and rat brain lysates (Figure 1D). The high degree of conservation is further supported by our prior study, which demonstrated that the N-terminal domain of lamprey γ-synuclein can also bind avidly to small lipid vesicles *in vitro,* like human α-synuclein (Busch et al., 2014). Moreover, when introduced in excess at stimulated lamprey synapses, recombinant lamprey γ-synuclein, its N-terminal domain, and human α-synuclein all phenocopied each other and inhibited SV endocytosis (Busch et al., 2014). Thus, the current data indicate that lamprey γ-synuclein and human α-synuclein are highly conserved both structurally and functionally, though we acknowledge that the variation in amino acid sequence within the C-terminus may lead to some functional differences that are as yet to be determined.

### Acute Perturbation of Synuclein with a Pan-Synuclein Antibody Disrupts SV Clusters at Resting Lamprey Synapses

**FIGURE 2:**
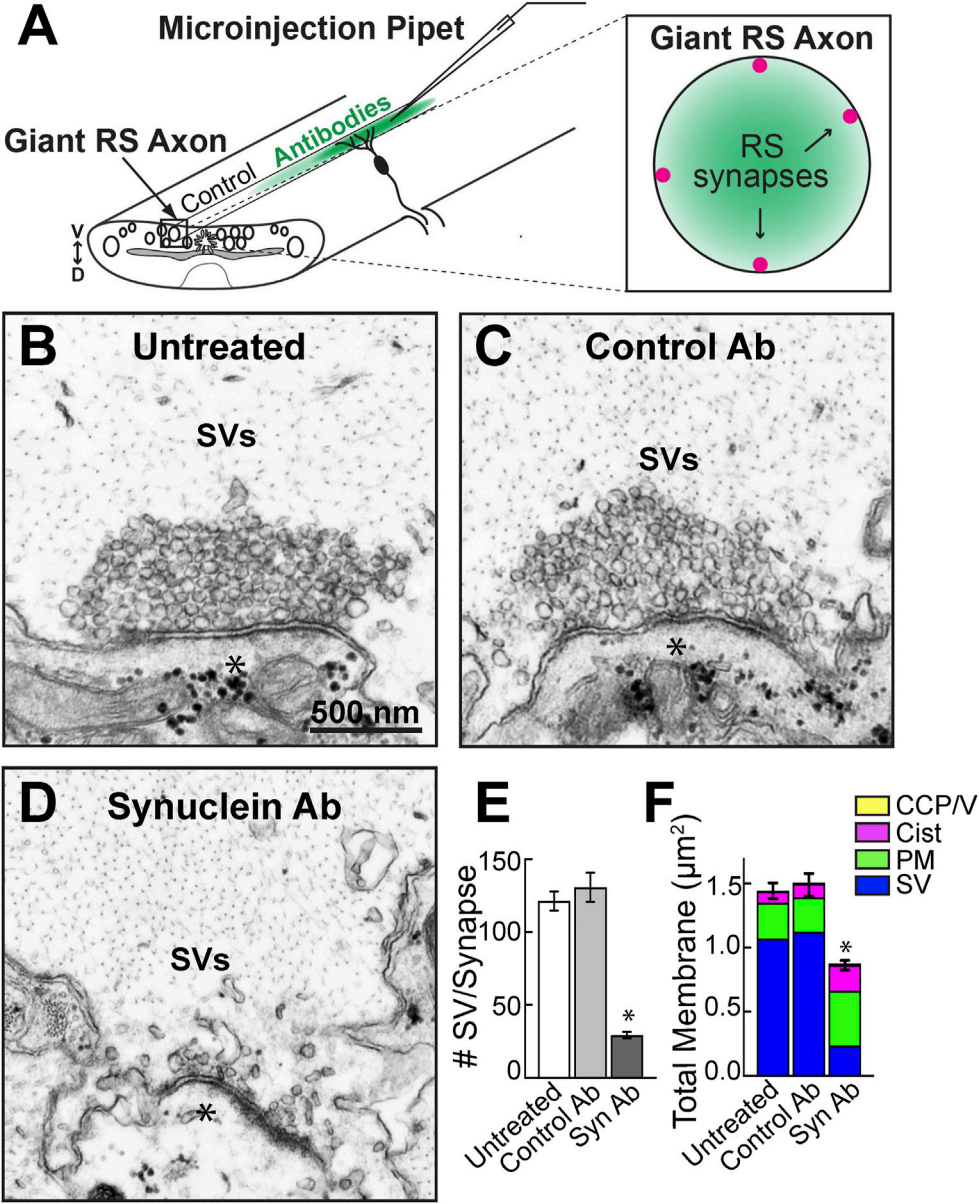
Microinjection of a pan-synuclein antibody induced a severe depletion of synaptic vesicles at resting lamprey synapses. **(A)** Diagram showing acute perturbation strategy. Antibodies are injected directly into the giant RS axons **(left),** which delivers the reagents directly to the synaptic vesicle clusters at resting RS synapses **(right).** Note the concentration gradient of injected antibodies, which permits evaluation of dose-dependent effects. **(B–D)** Electron micrographs of giant RS synapses. At untreated synapses, or after injection with Control IgG antibodies (Ab), the synaptic vesicle (SV) clusters are large and tightly clustered. After treatment with the Synuclein Ab, the SV clusters were severely depleted. Asterisks mark postsynaptic dendrites. Scale bar in B is 500 nm and applies to **C–D**. **(E)** Compared to controls, the synuclein antibody reduced the number of SVs in the vesicle cluster by >75%. Data are plotted as mean/section/synapse. **(F)** The synuclein antibody also significantly reduced the total amount of membrane at synapses, primarily due to the loss of membrane associated with SVs. Moderate increases in plasma membrane (PM) and cisternae (cist; putative endosomes) were observed. Bars in E and F represent mean ± SEM from n = 23–62 synapses, 2-5 axons/animals. Asterisks indicate statistical significance (*p* < 0.0001) by ANOVA, Tukey post hoc.

To determine whether synuclein regulates SV clustering *in vivo*, we acutely introduced the pan-synuclein antibody (ab6176; Abcam) to lamprey giant RS synapses via axonal microinjection (Figure 2A). We previously reported that this pan-synuclein antibody recognizes all three lamprey synucleins (Busch and Morgan, 2012). However, we predict that the antibody injections predominantly disrupt one of the γ-synuclein isoforms (GenBank: JN544525), since that is the only synuclein isoform expressed at appreciable levels within the lamprey giant RS neurons (Busch and Morgan, 2012).

RS synapses are *en passant*, glutamatergic synapses that reside along the perimeter of the giant RS axons within the ventral spinal cord. Axonal microinjection therefore delivers the antibody directly to the presynaptic SV clusters (Figure 2A). Within the dissected spinal cord, the giant RS axons and synapses are quiescent unless exogenously stimulated with action potentials. Thus, in the absence of stimulation, we can determine how different perturbations affect resting SV clusters at unstimulated synapses. Untreated, resting RS synapses possessed a large, tight cluster of SVs adjacent to the active zone (Figure 2B). Similarly, unstimulated synapses treated with Control IgG antibodies (Control Ab; Rabbit IgG isotype control; Thermo Fisher) also exhibited large SVs clusters without any noticeable changes in morphology (Figure 2C). In contrast, the pan-synuclein antibody (Synuclein Ab; ab6176; Abcam) severely disrupted the SV clusters, leaving only a few vesicles around the active zone (Figure 2D). Quantitative analysis confirmed that treatment with the synuclein antibody caused a >75% reduction in the number of SVs at resting synapses (Figure 2E; Untreated: 121 ± 7 SVs, *n* = 62 synapses, 5 axons; Control Ab: 130 ± 10 SVs, *n* = 29 synapses, 3 axons; Syn Ab: 29 ± 2 SVs, n = 23 synapses, 2 axons; ANOVA, *p* < 0.0001, Tukey’s *post hoc*). Although the remaining SVs sometimes appeared misshapen, their mean diameters were not significantly altered (Untreated: 51.5 ± 0.6 nm; Syn Ab-Low 50.6 ± 0.7 nm; Syn Ab-High 49.2 ± 0.8 nm; *n* = 206–209 SVs, 23–31 synapses; ANOVA *p* = 0.90; Tukey’s post hoc).

To determine whether the SV membrane was redistributed to other synaptic compartments, such as the plasma membrane, cisternae (putative endosomes), and/or clathrin-coated pits or vesicles (CCP/Vs), we performed a total membrane analysis for each synapse. The dramatic reduction of SV membrane was only partially compensated by modest increases in plasma membrane evaginations and cisternae, resulting in a 40% net loss of total membrane at synapses (Figure 2F; Untreated: 1.44 ± 0.06 μm^2^, n = 62 synapses, 5 axons; Control Ab: 1.50 ± 0.09 μm^2^, *n* = 29 synapses, 3 axons; Syn Ab: 0.87 ± 0.04 μm^2^, n = 23 synapses, 2 axons; ANOVA, *p* < 0.0001, Tukey’s *post hoc*). Thus, acute disruption of synuclein function at resting synapses induced a severe depletion of SVs at the active zone. The loss of total membrane suggests that the SVs escaped away from the immediate synaptic area.

### Introduction of SV2 or VAMP Antibodies do Not Affect the SV Clusters at Resting Lamprey Synapses

**FIGURE 3:**
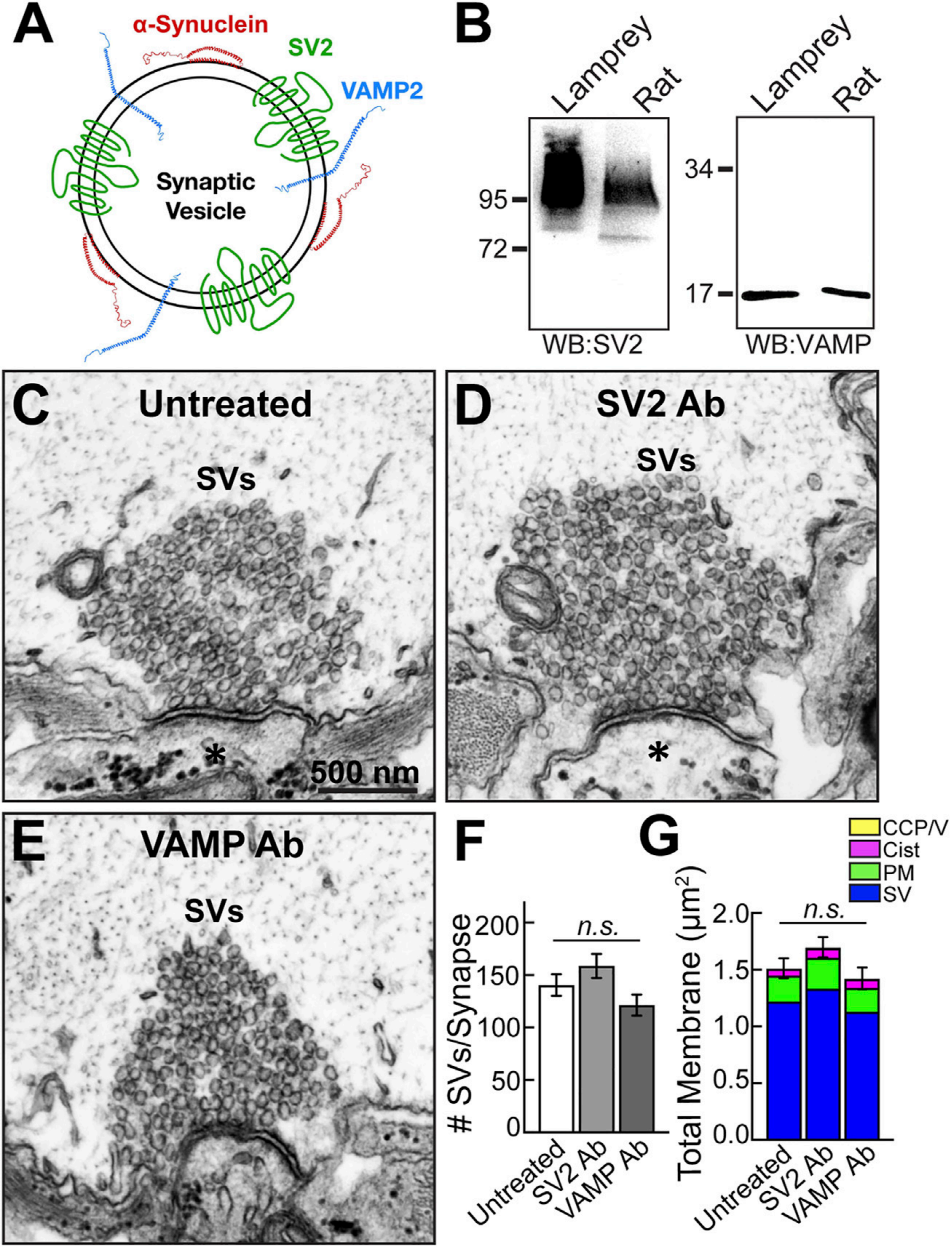
Microinjection of SV2 and VAMP antibodies had no noticeable effect on synaptic vesicle clusters. **(A)** Diagram of a synaptic vesicle showing α-synuclein and two other vesicle-associated proteins, SV2 and VAMP. **(B)** Western blots against SV2 and VAMP show bands of the expected molecular weights in both lamprey CNS and rat brain protein lysates. The SV2 band appears as a smear due to extensive glycosylation of the protein. **(C–E)** Compared to untreated synapses, no apparent changes in the morphologies of synaptic vesicle clusters were observed after treatment with either the SV2 or VAMP antibody. Asterisks mark the postsynaptic dendrites. Scale bar in C is 500 nm and applies to D-E. **(F)** SV2 and VAMP antibodies did not significantly impact the number of synaptic vesicles at synapses. Data are plotted as mean/section/synapse. **(G)** No changes were observed in the membrane distributions after treatment with the SV2 or VAMP antibody. Bars in F and G represent mean ± SEM from *n* = 24–38 synapses, 2-4 axons/animals. *n. s.* indicates “not significant” by ANOVA.

To determine the specificity of the phenotype produced by the synuclein antibody, we also injected RS axons with antibodies raised against two other abundant SV proteins, synaptic vesicle glycoprotein 2 (SV2) and vesicle-associated membrane protein (VAMP/synaptobrevin) (Figure 3A). Like synapsin, VAMP2 is another known binding partner of α-synuclein (Burre et al., 2010; Diao et al., 2013; Sun et al., 2019). The SV2 antibody (DSHB) is a mouse monoclonal raised against synaptic vesicles purified from the electric ray (*Discopyge ommata*), and it recognizes synaptic vesicle clusters in all vertebrates tested, including lampreys (Buckley and Kelly, 1985; Lau et al., 2011; Busch and Morgan, 2012; Busch et al., 2014). Previous studies using immunofluorescence and immunogold EM techniques showed that this SV2 antibody strongly labels the SV clusters at resting lamprey giant synapses, demonstrating that it penetrates the SV cluster and reaches accessible epitopes (Bloom et al., 2003; Busch et al., 2014; Banks et al., 2020). The VAMP antibody (1933-SYB/SP-10; PhosphoSolutions) is a mouse monoclonal raised against crude synaptic immunoprecipitate from human brain. As shown by Western blotting, these antibodies recognized protein bands of the predicted molecular weights for SV2 (95 kDa) and VAMP (∼16 kDa) in both lamprey CNS and rat brain lysates (Figure 3B).

Compared to untreated control synapses, resting synapses treated with either the anti-SV2 antibody or anti-VAMP antibody had a normal appearance with a large pool of tightly clustered SVs, and no disruptions were detected (Figures 3C–E). There was no significant difference in the number of SVs after treatment with either the SV2 or VAMP antibody (Figure 3F; Untreated: 140 ± 10 SVs, *n* = 38 synapses, 4 axons; SV2 Ab: 158 ± 11 SVs, *n* = 24 synapses, 2 axons; VAMP Ab: 121 ± 10 SVs, *n* = 25 synapses, 2 axons; ANOVA, *p* = 0.085). Likewise, there was no change in the total membrane or any of the individual synaptic compartments (i.e., SVs, PM, cisternae, CCP/Vs) (Figure 3G; Untreated: 1.51 ± 0.09 μm^2^, *n* = 38 synapses, 4 axons; SV2: 1.69 ± 0.14 μm^2^, *n* = 24 synapses, 2 axons; VAMP: 1.42 ± 0.10 μm^2^, *n* = 25 synapses, 2 axons; ANOVA, *p* = 0.147). Therefore, the loss of synaptic vesicles observed with the pan-synuclein antibody was specific to disrupting synuclein and not simply a non-specific phenotype induced by interfering with any SV-associated protein.

### The Pan-Synuclein Antibody Induces a Dose-Dependent Loss of SVs From the Cluster

**FIGURE 4:**
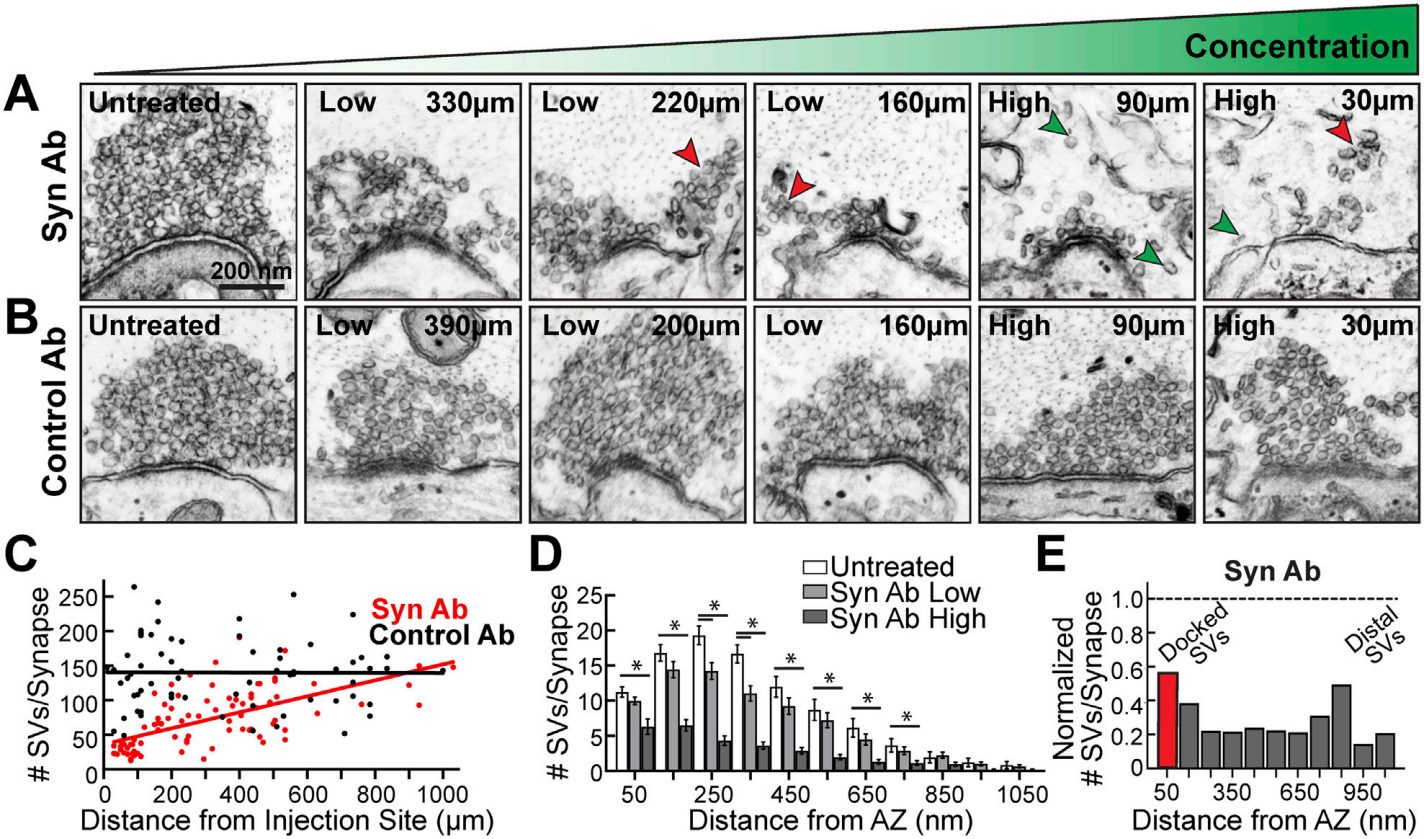
Introduction of the pan-synuclein antibody caused a dose-dependent loss of synaptic vesicles. **(A–B)** Electron micrographs showing synaptic vesicle clusters at lamprey RS synapses treated either with the synuclein antibody (Syn Ab) or Control IgG antibodies (Control Ab). Distances from the injection site are indicated. While Synuclein Ab caused a progressive loss of SVs as antibody concentration increased, there was no change in the size of vesicle clusters with the Control Ab. In the presence of the Synuclein Ab, the SVs appeared to de-cluster into smaller SV subclusters (red arrowheads), as well as individual SVs (green arrowheads). Scale bar in A also applies to B. **(C)** With the Synuclein Ab, vesicle cluster sizes were dependent on the distance from the injection site, and thus antibody concentration, while no effect was observed with the Control Ab (Syn Ab: slope = 0.1158, R^2^ = 0.394; Control Ab: slope = −0.0011, R^2^ < 0.0001; linear regression, *p* < 0.0001). **(D)** There was a dose-dependent effect of Synuclein Ab at all distances from the active zone (AZ) up to 800 nm. **(E)** Normalized vesicle distribution, which compares Synuclein Ab (High concentration) to untreated control, reveals that synuclein disruption caused a 50–80% loss of both docked synaptic vesicles (<50 nm, red bar), as well as the reserve pool of distal SVs (>50 nm, Gy bars). Bars in **D–E** represent mean ± SEM from *n* = 23–31 synapses, 2 axons/animals. Asterisks indicate statistical significance by ANOVA.

We next examined whether there was a dose-dependent effect of the synuclein antibody. Axonal microinjection results in a concentration gradient of synuclein antibody down the axon, due to lateral diffusion, with the highest concentration around the injection site (see Figure 2A). Based on the diffusion pattern of the co-injected fluorescent dye, we categorized synapses as receiving a “high” concentration (20–140 μm from the injection site) or “low” concentration of antibody (150–390 μm from the injection site). At distances farther from the injection site (>400 μm), the synapses received no antibody, thus providing untreated synapses as an internal control. Indeed, the pan-synuclein antibody induced a dose-dependent loss of SVs with increasing antibody concentration (Figure 4A). At many synapses, smaller subclusters of SVs were observed detaching from the main vesicle cluster, suggesting that synaptic vesicles were de-clustering into smaller units (Figure 4A, red arrowheads). De-clustering of individual SVs was also observed (Figure 4A, green arrowheads). In contrast, after injection of the Control IgG antibodies, the SV clusters remained large and tightly clustered at all distances evaluated (Figure 4B). At synapses treated with the synuclein antibody, the SV cluster sizes were positively correlated with distance from the injection site, indicating a dose-dependent response, whereas this was not observed with the Control antibodies (Figure 4C) (Synuclein Ab: slope = 0.1158, R^2^ = 0.394; Control Ab: slope = -0.0011, R^2^ < 0.0001; linear regression, *p* < 0.0001). This dose-dependence was also apparent in the binned distribution of SVs (Figure 4D) (two-way ANOVA, *p* < 0.01, Tukey’s *post hoc*). Synuclein perturbation with the pan-synuclein antibody (high concentration) induced a ∼50–80% loss of SVs at all distances evaluated (Figure 4E). This included a loss of the docked SVs closest to the active zone (Figure 4E). Synuclein is therefore necessary for proper SV clustering at synapses, including the maintenance of docked SVs and the distal reserve pool.

### Synuclein Perturbation Causes SV Dispersion and De-Clustering

**FIGURE 5:**
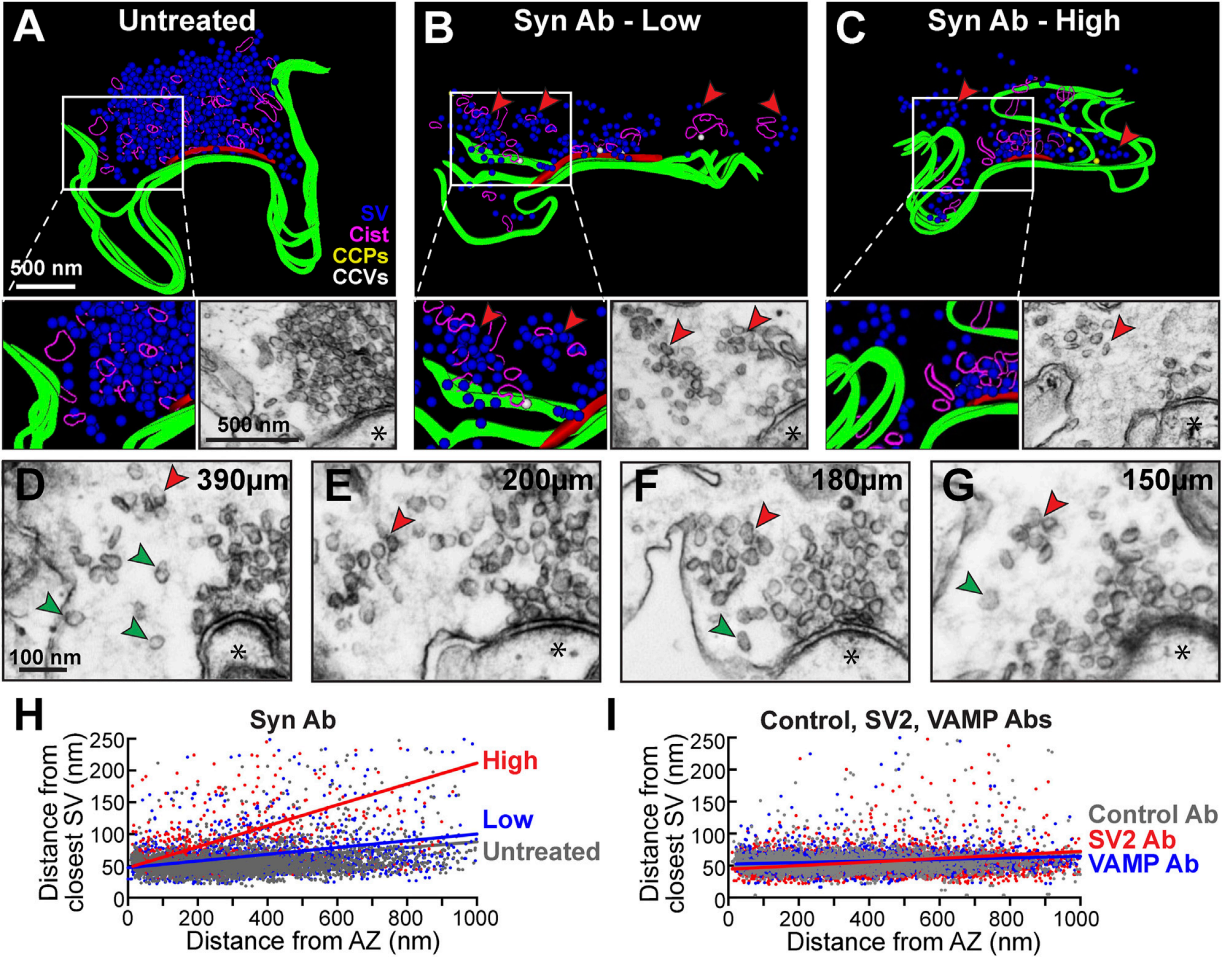
Inhibition of synuclein causes synaptic vesicle de-clustering at resting synapses. **(A–C)** 3D reconstructions of Untreated synapses and those treated with Synuclein antibody (low and high concentrations). Inhibiting synuclein function causes SVs to de-cluster into smaller clumps (B-C insets, red arrowheads). Scale bar in A applies to **(B–C**). **(D–G)** Electron micrographs showing additional examples of SV de-clustering into SV subclusters (red arrowheads), as well as individual single SVs (green arrowheads). Asterisks indicate postsynaptic densities. Scale bar in D applies to **(E–H)**. **(H–I)** Nearest neighbor analysis revealed SV de-clustering after treatment with the synuclein antibody, especially at high concentrations. This was no observed with high concentrations of the Control, SV2, or VAMP antibodies, where most SVs remained tightly clustered within 50–70 nm away from their nearest neighbor. Data points are from *n* = 676–3793 SVs, 23–31 synapses, 2-3 axons/animals.

To further explore the phenotype, we generated 3D reconstructions of synuclein antibody-treated synapses. At untreated synapses, SVs are typically tightly clustered into a single, large SV cluster at the active zone (Figure 5A). Further supporting a role for synuclein in SV clustering, the pan-synuclein antibody-treated synapses often exhibited smaller, discrete SV subclusters, which were dispersed away from the active zone (Figures 5B,C, red arrowheads). These miniature SV subclusters were clearly separated from the main SV cluster and varied in size and shape (Figures 5B–G, red arrowheads). Single SVs were also observed as part of the de-clustering phenotype (Figures 5D–G, green arrowheads). To quantify the SV de-clustering, we performed a nearest-neighbor analysis, which measured the shortest distance between the center of each SV and its nearest neighbor. Synaptic vesicles that are tightly clustered together have a nearest neighbor within ∼50–60 nm, which is approximately the diameter of a synaptic vesicle at lamprey synapses (Medeiros et al., 2017; Román-Vendrell et al., 2021). As expected, the untreated synapses were tightly clustered (Figure 5H, gray). In contrast, synapses treated with the pan-synuclein antibody were de-clustered, most especially with the higher concentration of antibody (Figure 5H, blue and red). With the synuclein antibody, the nearest neighbor distances significantly increased, ranging from ∼50 nm close to the active zone to ∼200 nm farther from the active zone (Figure 5H, blue and red) (Untreated: slope = 0.0447, R^2^ = 0.0730, *n* = 2986 SVs, 30 synapses, 2 axons; Synuclein Ab Low: slope = 0.0530, R^2^ = 0.2440, *n* = 2418 SVs, 31 synapses, 2 axons; Synuclein Ab High: slope = 0.1645, R^2^ = 0.0805, *n* = 676 SVs, 23 synapses, 2 axons; linear regression; multiple comparisons *p* = 0.002). Thus, synuclein perturbation resulted in a de-clustering and dispersion of synaptic vesicles away from the active zone. In comparison, synapses treated with the higher concentrations of the Control, SV2, and VAMP antibodies remained on average ∼50–70 nm from their nearest neighbor, reflective of tight SV clustering (Figure 5I; Control IgG Ab: slope = 0.0222, *n* = 2928 SVs, 29 synapses, 3 axons; SV2 Ab: slope = 0.0289, *n* = 4079 SVs, 24 synapses, 3 axons; VAMP Ab: slope = 0.0122, *n* = 3032 SVs, 25 synapses, 2 axons; linear regression, multiple comparisons *p* = 0.028). Thus, synuclein regulates SV clustering at resting synapses, and disruption of synuclein function disperses SVs into both smaller discrete clusters and individual SVs.

## Discussion

**FIGURE 6:**
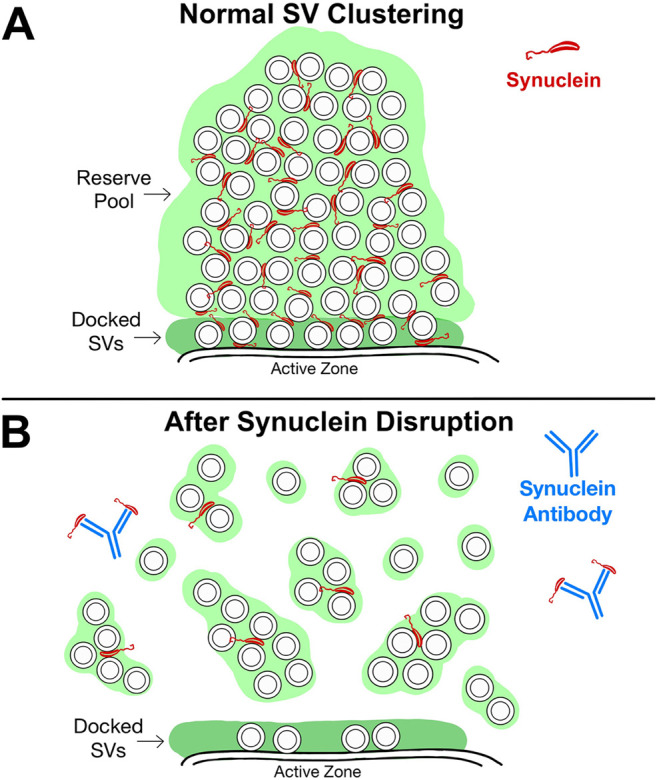
Working model for synuclein’s role in SV clustering. **(A)** At resting, intact synapses, synuclein is localized to the SV clusters. The distal reserve pool of SVs is denoted by the light green region, and the pool of docked SVs is denoted by the dark green region. **(B)** After synuclein disruption using the pan-synuclein antibody, SVs in the distal reserve pool disperse into smaller subclusters, as well as individual SVs. The remaining SV subclusters may be held together via synapsin-mediated LLPS or some other scaffolding mechanism involving protein tethers. After synuclein disruption, SVs nearest the active zone are also depleted, indicating a role for synuclein in SV docking.

To our knowledge, this is the first *in vivo* demonstration of a critical role for synuclein in SV clustering at a vertebrate synapse. Recent *in vitro* studies suggested a role for α-synuclein in vesicle clustering under reduced experimental conditions (Diao et al., 2013), perhaps in coordination with synapsin (Hoffmann et al., 2021). But the extent to which this occurs in the complex environment of a living synapse was unclear until now. Our experiments showed that microinjection of a pan-synuclein antibody led to a rapid, dose-dependent dispersion and de-clustering of synaptic vesicles at lamprey synapses, resulting in a loss of both docked synaptic vesicles, as well as the reserve pool (Figures 6A–B). We therefore propose that synuclein could work cooperatively with synapsin to maintain vesicle clusters, as has been suggested by a recent *in vitro* study (Hoffmann et al., 2021). Alternatively, the synuclein antibody may have disrupted the SV clusters by interfering with synuclein’s interactions with other cytosolic and/or SV-associated proteins, such as cytoplasmic dynein, chaperones, and/or other SV-associated binding partners (Chu et al., 2012; Longhena et al., 2019; Banks et al., 2020). Interestingly, after synuclein antibody treatment, the synaptic vesicles appeared to disperse into smaller SV subclusters (Figure 6B), which may remain held together via synapsin-mediated LLPS or some other mechanism involving protein tethers (Zhang and Augustine, 2021). Although we do not yet know the precise mechanisms, current data suggest that synuclein may function to cross-link the smaller SV clumps together, thereby assembling them into larger SV clusters at the active zone. In this respect, our data are consistent with a hybrid view that incorporates both the “scaffolding” and LLPS models.

In addition to the loss of SVs, the pan-synuclein antibody also caused a modest but significant increase in the plasma membrane evaginations and cisternae, which are likely some type of endosomes (Figure 2F). The most plausible explanation is that inhibiting endogenous synuclein somehow aberrantly stimulated exocytosis, which is consistent with the known role for synuclein in regulating the kinetics of synaptic vesicle exocytosis (Logan et al., 2017). However, additional follow up experiments using electrophysiological measurements would be necessary to unequivocally determine whether this is the case.

Strictly speaking, data presented here demonstrate a role for lamprey synuclein in SV clustering and SV docking, and the extent to which this applies to other synuclein orthologs from other vertebrate species remains to be seen. However, given that the inhibitory antibody was directed against an N-terminal domain epitope that is highly conserved in all synuclein orthologs ranging from lampreys to humans (Figure 1) suggests that the SV clustering and docking functions will be more broadly ascribed to other synuclein isoforms as well (Busch and Morgan, 2012). The fact that human α-synuclein can cluster SV-like vesicles *in vitro* provides some corroborating evidence (Diao et al., 2013; Hoffmann et al., 2021), as does the phenotypic overlap between lamprey and mammalian synucleins observed at stimulated synapses (Nemani et al., 2010; Busch et al., 2014). However, to definitively establish whether or not the SV clustering and docking functions are more broadly shared amongst synuclein family members will require additional testing, since other synaptic functions of α−, β−, and γ-synucleins (e.g., those ascribed to the C-terminus) are not fully redundant (Ninkina et al., 2012; Somayaji et al., 2020).

Ultimately, our findings that synuclein regulates SV clustering and docking expand the growing list of synuclein’s functions at synapses, which also include regulation of SNARE complex formation (Burre et al., 2010), exocytosis and fusion pore dilation (Logan et al., 2017), vesicle endocytosis (Busch et al., 2014; Vargas et al., 2014; Xu et al., 2016; Eguchi et al., 2017), and activity-dependent vesicle re-clustering after endocytosis (Nemani et al., 2010). Thus, synuclein appears to be a multi-functional protein that modulates many stages of synaptic vesicle trafficking. It is likely that there are stage-dependent interactions between synuclein and other presynaptic binding partners. Understanding the temporal dynamics and specific functions of these interactions should be a priority for future studies.

Interestingly, a previous study reported that synaptic vesicle clusters at α/β/γ-synuclein knockout synapses were more densely packed and tightly clustered (Vargas et al., 2017), which may at first appear contradictory to our results. However, it is well established that genetic manipulation of synuclein leads to compensatory changes in expression levels for synapsin and several other key presynaptic proteins (Nemani et al., 2010; Scott et al., 2010), as well as altered levels of synapsin phosphorylation (Vargas et al., 2017). Specifically, triple knockout of α/β/γ-synuclein leads to overexpression of synapsin (Greten-Harrison et al., 2010), which may explain the tightly clustered SVs at synapses (Vargas et al., 2017). Conversely, chronic overexpression of α-synuclein causes downregulation of synapsin protein expression, including at synapses (Nemani et al., 2010; Scott et al., 2010), which is coincident with less dense SV clusters as would be predicted by the lower levels of synapsin (Scott et al., 2010; Fornasiero et al., 2012; Orenbuch et al., 2012; Vargas et al., 2017). This example demonstrates a strong genetic interaction between α-synuclein and synapsin, which has now been corroborated by a direct interaction and functional studies at synapses (Atias et al., 2019). What is not yet clear is why chronic α-synuclein overexpression and acute perturbation of endogenous synuclein both lead to SV dispersion phenotypes, emphasizing the importance of determining the compensatory molecular changes that occur alongside any synuclein-targeted perturbations.

Since the inhibitory antibody is directed toward the N-terminus of synuclein, this region of the protein is likely important for SV clustering and docking. The N-terminal domain of α-synuclein, and other synuclein orthologs including lamprey γ-synuclein, mediates membrane binding (Davidson et al., 1998; Chandra et al., 2003; Burre et al., 2012; Busch et al., 2014). We therefore propose that membrane binding is essential for synuclein’s function in synaptic vesicle clustering, as it is in other synaptic vesicle trafficking events (Nemani et al., 2010; Busch et al., 2014). Although we do not know the specific conformation(s) of endogenous synuclein present at synapses, a model involving the broken helix conformation is attractive, due to the possibility of making linkages between synaptic vesicles (e.g., within the reserve pool), as well as linking SVs to the plasma membrane (i.e., the docked synaptic vesicles) (Chandra et al., 2003; Snead and Eliezer, 2019).

At least one key difference exists between the phenotype produced by acute disruption of synuclein function at lamprey synapses and that which occurs after acutely perturbing synapsin function. Disruption of synapsin function with antibodies directed against the intrinsically-disordered region led to a selective loss of the distal pool of synaptic vesicles at lamprey synapses, consistent with the reserve pool, leaving the docked synaptic vesicles at the active zone relatively intact (Pieribone et al., 1995; Pechstein et al., 2020). In contrast, acutely inhibiting synuclein function reduced both the reserve pool, as well as the number of docked synaptic vesicles (Figure 6B). This indicates that synuclein has a functional role *in vivo* in docking synaptic vesicles to the active zone, which is consistent with recent *in vitro* data (Lou et al., 2017; Man et al., 2021). Synuclein-dependent synaptic vesicle docking could be mediated through its interaction with VAMP2 and the SNARE complex, as well as through phospholipids enriched in the active zone membrane (Burre et al., 2010; Diao et al., 2013; Lou et al., 2017; Sun et al., 2019).

Going forward, it will be necessary to gain a better understanding of the underlying mechanisms by which α-synuclein regulates synaptic vesicle clustering and docking, and the extent to which these functions occur through its interactions with other presynaptic proteins such as synapsin and VAMP2 (Burre et al., 2010; Atias et al., 2019; Sun et al., 2019; Hoffmann et al., 2021). Here is another place where acute perturbations at large vertebrate synapses such as the lamprey reticulospinal synapse and mammalian calyx of Held could be particularly useful at providing initial mechanistic insights, as has been the case for several decades of study on α-synuclein, synapsin and other critical presynaptic proteins (Pieribone et al., 1995; Ringstad et al., 1999; Brodin and Shupliakov, 2006; Busch et al., 2014; Xu et al., 2016; Eguchi et al., 2017; Medeiros et al., 2017; Banks et al., 2020; Pechstein et al., 2020; Román-Vendrell et al., 2021). Such insights will have significant implications for understanding α-synuclein-associated diseases that affect synapses, including Parkinson’s disease, dementia with Lewy bodies, and other related synucleinopathies.

## Data Availability

The raw data supporting the conclusions of this article will be made available by the authors, without undue reservation.
